# Arachnids of medical importance in Brazil: main active compounds present in scorpion and spider venoms and tick saliva

**DOI:** 10.1186/s40409-015-0028-5

**Published:** 2015-08-13

**Authors:** Francielle A. Cordeiro, Fernanda G. Amorim, Fernando A. P. Anjolette, Eliane C. Arantes

**Affiliations:** Department of Physics and Chemistry, School of Pharmaceutical Sciences of Ribeirão Preto, University of São Paulo (USP), Avenida do Café, s/n, Ribeirão Preto, SP 14.040-903 Brazil

**Keywords:** Arachnid toxins, Scorpion venom, Spider venom, Tick saliva

## Abstract

Arachnida is the largest class among the arthropods, constituting over 60,000 described species (spiders, mites, ticks, scorpions, palpigrades, pseudoscorpions, solpugids and harvestmen). Many accidents are caused by arachnids, especially spiders and scorpions, while some diseases can be transmitted by mites and ticks. These animals are widely dispersed in urban centers due to the large availability of shelter and food, increasing the incidence of accidents. Several protein and non-protein compounds present in the venom and saliva of these animals are responsible for symptoms observed in envenoming, exhibiting neurotoxic, dermonecrotic and hemorrhagic activities. The phylogenomic analysis from the complementary DNA of single-copy nuclear protein-coding genes shows that these animals share some common protein families known as neurotoxins, defensins, hyaluronidase, antimicrobial peptides, phospholipases and proteinases. This indicates that the venoms from these animals may present components with functional and structural similarities. Therefore, we described in this review the main components present in spider and scorpion venom as well as in tick saliva, since they have similar components. These three arachnids are responsible for many accidents of medical relevance in Brazil. Additionally, this study shows potential biotechnological applications of some components with important biological activities, which may motivate the conducting of further research studies on their action mechanisms.

## Background

Envenomings are considered a neglected disease by the World Health Organization [1] and constitute a public health problem, especially in tropical countries. The animals responsible for such accidents possess an apparatus associated with a venom gland that is able to produce a mixture rich in toxic and nontoxic components [2]. Among the most studied arthropod venoms are those from scorpions, spiders and ticks, belonging to the phylum Arthropoda, class Arachnida, which correspond to the purpose of this review. They are widely dispersed in urban centers due to the large availability of shelter and food, which facilitates their reproduction and consequently increases the number of accidents [3, 4]. Therefore, this review will focus on the main Brazilian venomous animals of the Arachnida class belonging to Scorpionida, Araneae, Ixodidae orders as well as on the aspects related to envenoming caused by these animals and their venom/saliva composition, highlighting the components of scientific and medical interest.

**Fig. 1:**
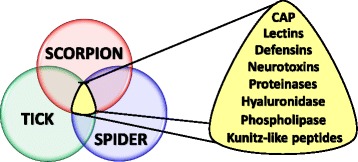
Venn diagram highlighting the protein families presented in tick saliva and scorpion/spider venoms. Catabolite activator protein (CAP), defensins, hyaluronidase, Kunitz-like peptides (serine proteinase inhibitor), neurotoxins, lectins and phospholipase are some of the compounds shared among these arthropods

The phylogenomic analysis of the nuclear protein-coding sequences from arthropod species suggests a common origin in the venom systems of scorpions, spiders and ticks [5, 6]. Specifically, catabolite activator protein (CAP), defensins, hyaluronidase, Kunitz-like peptides (serine proteinase inhibitor), neurotoxins, lectins and phospholipase are examples of compounds shared by these animals (Fig. 1). Some compounds such as alanine-valine-isoleucine-threonine protein (AVIT protein) and sphingomyelinase have been identified in spiders and ticks. Cystatins, lipocalins and peptidase S1 are found only in ticks [5].

In this context, the study of the structural similarity among these compounds/toxins identified in the venom/saliva of these animals may contribute to a better understanding of the action mechanism involved in envenoming besides providing information about molecules with great biotechnological potential.

## Review

### Scorpion venoms

Scorpion envenoming is considered a public health problem, especially in tropical countries [7]. Annually, more than one million cases of scorpion envenomation are reported worldwide with a fatality risk of around 3 % [8]. According to the data from the Brazilian Ministry of Health, 57,933 accidents were recorded in Brazil in 2011, of which 91 cases resulted in death [9].

**Fig. 2:**
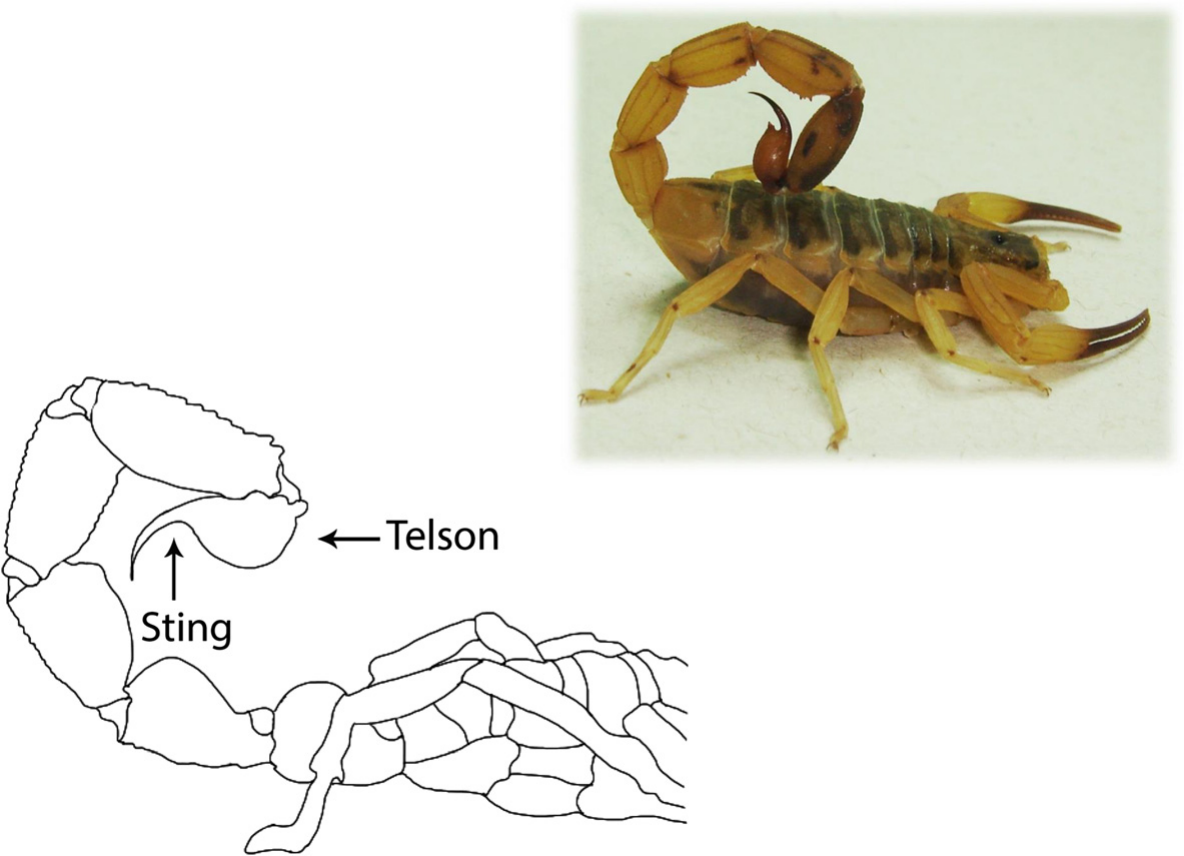
Photo of a scorpion and schematic representation of scorpions’ telson. Morphology of the inoculum apparatus of scorpion venom located on the last segment of the post-abdomen of the animal. The telson comprises a pair of glands responsible for the production and storage of the venom used for feeding and self-defense of the scorpion

The scorpion venom apparatus consists of a gland connected to a telson sting which is located on the last segment of the post-abdomen of the animal (Fig. 2). This is an apparatus of great importance for their survival, assisting in feeding and self-defense of the scorpion. The telson has a vesicle that contains a pair of glands responsible for the production and storage of the venom [2].

A scorpion sting is characterized by intense pain and systemic symptoms that usually develop rapidly [10]. According to clinical manifestations, scorpion envenomings are classified as mild, moderate or severe. The general initial response to a scorpion sting is immediate local burning pain, which may be severe. General symptoms may occur soon after the sting, but may be delayed for several hours. Therefore, vital functions of patients with systemic manifestations should be observed continuously, while seeking early treatment of the complications [11].

So far, approximately 2,000 species of scorpions have been described, distributed worldwide. These arachnids are classified into seven families: Scorpionidae, Diplocentridae, Chactidae, Vaejovidae, Bothriuridae, Chaerilidae and Buthidae. The most dangerous species belong to the family Buthidae, which comprises more than 500 species. In Brazil the scorpions with the highest medical and scientific interest belong to the genus *Tityus* [2, 12–15].

There are more than ten different *Tityus* species in Brazil, among which *Tityus stigmurus*, *Tityus bahiensis* and *Tityus serrulatus* are primarily responsible for human envenoming. *T. serrulatus* is considered the most dangerous species in the country, responsible for the highest number of envenoming accidents [16, 17].

### Biochemical characteristics of the venom from *Tityus*

Scorpion venoms are a complex mixture of substances that include: inorganic salts, free amino acids, heterocyclic components, peptides and proteins, mainly enzymes that are used by the scorpions for self-defense and the capture of prey [18]. A broad range of bioactive compounds of scorpion venoms have already been purified and characterized. It is estimated that the number of different components present in these venoms is approximately 100,000, but only 1 % of these molecules have been isolated and characterized [19]. The advent of recombinant DNA technology, such as transcriptome analysis, allowed the identification of new components; however, some of them have not yet been directly purified from the venom.

Venoms varies compositionally from genus to genus and species to species and may differ in potency, probably due to changes in the proportion of their toxins, associated with genetic and environmental variations, such as diet and climate [20–23]. Studies have shown that *T. serrulatus* venom is two to three times more toxic than that of *T. bahiensis*, which explains the various studies that aimed to isolate and characterize their toxins [2]. Furthermore, such studies found variability in venom lethality among *T. serrulatus* specimens, which suggests that neurotoxins, such as α-type neurotoxin, must be the major lethal component in the whole venom [24].

The major components of scorpion venom are neurotoxins, which act on ion channels of excitable cells [25]. The venom compounds may interact with each other to modulate the function of ion channels, which is usually responsible for the known symptoms of envenoming. Scorpion neurotoxins present a tightly tridimensional-shaped backbone stabilized by three or four disulfide bridges. This property avoids their *in-vivo* degradation, thereby increasing their interaction time with ion channels and their efficacy [18].

Four different families of neurotoxins are usually found in scorpion venom: peptides that modulate sodium-, potassium-, chloride- or calcium-gated channels [12]. The most studied families of venom neurotoxins from *Tityus* species act on sodium and potassium channels. The poorly known toxins specific for chloride and calcium channels present variable amino acid lengths [26]. The neurotoxins present a highly conserved essential three-dimensional structure comprising an α-helix and three- or four-stranded anti-parallel β-sheets connected by two to four disulfide bonds [18, 27, 28].

The scorpion toxins that affect mammalian voltage-gated Na^+^ channels (Nav) are classified as: α-neurotoxins (α-NaScTx) and β-neurotoxins (β-NaScTx). The α-NaScTx interacts with channel receptor site 3 located in the S3–S4 extracellular loop in domain IV and in the S5–S6 extracellular linker domain I of Nav channels [2, 18]. The α-NaScTx retards the mechanism of Nav inactivation and prolongs the repolarization phase of the membrane action potential [2]. The α-NaScTx can be subdivided into the following three main groups: (1) classical α-toxins, which are highly active only in mammalian Nav channels and present poor toxicity against insects; (2) anti-insect α-NaScTXs, which are highly active only on insect Nav channels; and (3) α-like toxins, active on both insect and mammalian Nav channels [18]. As shown in Table 1, toxins such as Ts3 isolated from *T. serrulatus*, TbTx5 from *T. bahiensis* and Tst3 from *T. stigmurus* are highly conserved between the species sharing a high percentage of identity [29–31]. Those toxins also show high similarity with Ts5 of *T. serrulatus* and Tb3 of *T. bahiensis*. The Ts3 relaxes the human corpus cavernosum *in vitro* through the release of NO from nitrergic nerves and the elucidation of its action mechanism would be useful for the development of new therapeutic strategies to treat priapism after scorpion envenomation. Additionally, this is a molecule that can be used as a model for the development of a new drug to treat erectile dysfunction [32].

**Table 1:** Examples of compounds from *Tityus* scorpion venoms

Compounds	Examples	Species	Molecular Mass (kDa)	Action Mechanism	References
Neurotoxins	Ts3, Ts5	*Tityus serrulatus*	~6.0–7.0	Action on Na^+^ channels	29–32
	TbTx5, Tb3	*Tityus bahiensis*			
	Tst3	*Tityus stigmurus*			
	Ts1	*Tityus serrulatus*	6890.9		33–34
	Ts6, Ts7	*Tityus serrulatus*	~6.0–7.0	Action on K^+^ channels	35–40
	Tst26	*Tityus stigmurus*			
	Tt28	*Tityus trivittatus*			
	TdK1	*Tityus discrepans*			
Hypotensive agent	Hypotensin	*Tityus serrulatus*	2.75	Agonist of the B(2) receptor	41
Antimicrobial peptides	TsAP1, TsAP2	*Tityus serrulatus*	~8.4	Unclear	42
Proteinases	Metalloproteinase	*Tityus serrulatus*	~25.0	Lysis of the cell basement membrane	43–46
	Serine proteinases^a^	*Tityus serrulatus Tityus bahiensis*	–	Action on coagulation factors	47
Enzymes	Phospholipase^b^	*Tityus serrulatus Tityus stigmurus*	–	Hydrolysis of membrane phospholipids	48–49
	Hyaluronidase	*Tityus* sp*.*	~50.0	Catalyzes the hydrolysis of hyaluronan from the extracellular matrix	50

^a^Identified in the venom, but not purified

^b^Compound found only in the transcriptome

Another class of toxins that affect Nav channels is the β-neurotoxins (β-NaScTx), which bind to receptor site 4 in the extracellular loops that connect transmembrane segments S3 and S4 and the S1 and S2 segments in domain II [2, 18]. Thus, this class alters the voltage-dependence of channel activation to more negative potentials to cause an increased tendency to trigger the spontaneous and the repetitive potentials of the membrane [2]. Similar to α-NaScTx, the β-neurotoxins are subdivided into four groups according to their pharmacological selectivity for insect and mammalian Nav channels: (1) βm, active on mammalian Nav channels; (2) βi, selectively active on insect Nav channels; (3) β-like, for toxins without preference between mammalian and insect Nav channels and (4) β_α_, for those that presents a primary structure of β-toxins, but with a functional α-effect [14]. The toxin Ts1, a β-neurotoxin with action on Nav channels, is the most abundant toxin in *T. serrulatus* venom, whose activities include inducing macrophage activation *in vitro* [33, 34].

The neurotoxins that act on voltage-gated K^+^ channels (Kv) can be classified into α, β, γ and κ [35, 36]. There are two main types of structural motifs observed in these peptide classes: (1) the common motif comprised of one or two short α-helices connected to a triple-stranded antiparallel β-sheet stabilized by three or four disulfide bonds, denominated CS αβ and (2) the α-helix-loop-helix (CS αα) fold consisting of two short α-helices connected by a β-turn; only the kappa toxins adopt this fold [18, 37–40]. The α-neurotoxins (α-KTx) block the pore binding to the external vestibule of the channel and block the ionic conductivity by occlusion of the physical pore without affecting the kinetics of channel activation [41]. Ts6 and Ts7 from *T. serrulatus*, Tst26 from *T. stigmurus*, Tt28 from *T. trivittatus* and TdK1 from *T. discrepans* are examples of α-neurotoxins that act on Kv channels [35, 42–45].

In addition to α-KTxs, the venoms of the Buthidae, Caraboctonidae and Scorpioninae families also contain β-neurotoxins (β-KTxs) [35]. According to the identity of the sequences, these toxins may be divided into three classes. Class 1 containing the toxins TsTX-Kβ-related peptides, such as TsTx-Kβ, TtrβKTx, TdiβKTx, TstβKTx, Tco 42.14 from *T. serrulatus*, *T. trivittatus*, *T. discrepans*, *T. stigmurus* and *T. costatus*, respectively. The only peptide characterized to any extent is TsTx-Kβ from *T. serrulatus*, which is a blocker of Kv1.1 channel with IC50 values of 96 nM [46]. Class 2 consisting of peptides homologous to BmTXKβ from *Buthus martensii* which showed an inhibition of the transient outward K^+^ current (Ito) of rabbit atrial myocytes; some examples of class 2 peptides are TdiKIK, TtrKIK, TcoKIK and TstKMK [18]. Class 3 is formed by the Scorpine-like peptides, also known as “orphan” peptides. They possess two structural and functional domains: an N-terminal α-helix (with cytolytic and/or anti-microbial activity such as insect defensins) and a tightly folded C-terminal region with a CS αβ motif, displaying Kv channel blocking activity. The scorpine homologs exhibit strong antimicrobial effects as well as cytolytic activity against eukaryotic cells and possible antimalarical activity [18, 46, 47].

The other subclasses of neurotoxins that act on Kv channels, such as γ and κ, are less studied. However the γ-KTxs neurotoxins were described as mainly targeting hERG channels and were found in scorpions of the genus *Centruroides, Mesobuthus* and *Buthus* [18, 36]. The κ-KTxs neurotoxins show an interaction with voltage-gated Kv channels similar to α-KTx toxins, presenting the lysine and aromatic/hydrophobic residue (functional dyad) that interact with the channel [18].

The diversity of toxins that target Kv channels with high affinity and selectivity provides a large number of molecular structures that can be considered for the development of therapeutic drugs for diseases such as cancer and autoimmune diseases, in which there is an overexpression of these channels [48]. For example, the HERG channels are associated with cell cycle and proliferation of several cancers; therefore, the use of HERG-specific blockers could inhibit the proliferation of tumor cells [18].

The scorpion venoms are composed of other peptides and proteins such as hyaluronidases, antimicrobial peptides, phospholipases, allergens, hypotensins and also proteinases, such as serine proteinases and metalloproteinases, among others. However, some of these molecules were not isolated from the scorpion venoms and were only identified in the venom gland transcriptome.

In addition to the neurotoxic effects induced by toxins acting on ion channels, a wide variety of actions of the venom components can be observed such as hypotensive and antimicrobial effects induced by TsHpt-I and scorpine, respectively. TsHpt-I, isolated from *T. serrulatus* venom, acts as an agonist of the B(2) receptor and does not inhibit angiotensin-converting enzyme [49]. As described above, the *Tityus* venom possesses a peptide called scorpine which presents an antimicrobial and antimalarial activity [47]. Recently, Guo *et al.* [50] identified two others antimicrobial peptides, TsAP1 and TsAP2, with broad spectrum antimicrobial and anticancer activities. The antimicrobial peptides are cationic and amphipathic, mostly within 50 amino acid residues, were gathered into different groups and their action mechanisms remain unclear [12].

Although the presence of phospholipase was reported in the transcriptome of *T. serrulatus* and *T. stigmurus,* venoms of *T. serrulatus*, *T. bahiensis* and *T. stigmurus* exhibit significant proteolytic but no phospholipase activity [51–53]. The venom of these scorpions also showed metalloproteinase activity; however, this enzyme was obtained only from *T. serrulatus* venom [51, 54–56]. Furthermore, enzymes that present gelatinolytic activity, such as serine proteinases, were detected in *T. serrulatus* and *T. bahiensis* venoms, but these toxins have not been isolated yet [57].

Hyaluronidase, another important protein present in scorpion venom, is considered a “spreading factor” by favoring the absorption and spread of venom through the tissues of the victim, contributing to local or systemic envenoming [58]. Animals injected with Ts1, the major toxin from *T. serrulatus*, and hyaluronidase achieved significantly higher serum levels of creatine kinase (CK), lactate dehydrogenase (LD) and aspartate aminotransferase (AST) in a shorter time than those injected with only Ts1 (without hyaluronidase), confirming the characteristic of the “spreading factor” of the hyaluronidase. The animals, which received only hyaluronidase, showed CK, LD and AST levels similar to those of the control group, indicating no intrinsic toxic effect of hyaluronidase [59].

The advent of transcriptome analysis of the scorpion venom gland allowed the determination of several components that had not been purified from the venom of these animals. Transcriptome of several scorpions has been performed, and among the genus *Tityus* the transcriptomes of *T. stigmurus*, *T. discrepans*, *T. costatus* Karsch, *T. pachyurus, T. obscurus*, *T. bahiensis* and *T. serrulatus* have been reported [52, 53, 60–62]. These analyses found transcripts of novel proteins such as phospholipases, metalloproteinases, allergens, proteinases, antimicrobial peptides and anionic peptides. However, the possibility that those transcripts had undergone microRNA-mediated degradation during the processing period may explain why some toxins were found only in the transcriptome and not in the venom [53].

One of the major goals of the identification and characterization of animal toxins is the possibility of obtaining new therapeutic drugs. A famous example about scorpion toxins with biotechnological application is the chlorotoxin isolated from venom of the Israeli scorpion *Leiurus quinquestriatus*, which was initially developed for the diagnosis and treatment of glioma. Furthermore, this toxin was discovered to be capable of labeling specific cancer cells [63]. Although the biomarker responsible for the binding is still under discussion, it has been preliminarily identified as annexin 2A. Recently, the extremely stable iodinated analogue of this toxin—TM601, which presents no immunogenicity and produces no toxicity in humans—has successfully completed clinical phase II in the treatment of recurrent glioma and was approved by the Food and Drug Administration (FDA) [63–65].

Thus, given the wealth of components present in scorpion venom, it is concluded that the study of these toxins is not only a potential source of new drugs, but also a source of tools in the elucidation of the physiological systems and envenoming presented by these animals [66].

### Spider venoms

**Fig. 3:**
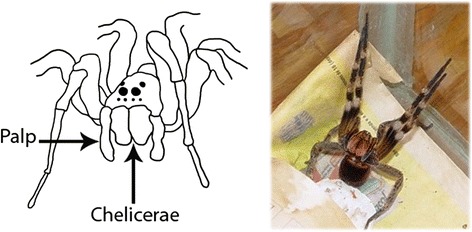
Photo of a spider and schematic representation of a spider’s chelicerae. Chelicerae are associated with venom glands, which are responsible for the production and storage of venom. The chelicerae are also used to trap and kill the prey

Spiders possess four pairs of paws and an external skeleton composed of chitin (Fig. 3). The exclusive feature of these animals is the presence of chelicerae associated with the venom gland, except for rare species. The spiders use their venom primarily to paralyze or kill their prey, sometimes for self-defense, which may cause occasional accidents [67].

The World Health Organization (WHO) establishes that only four spider genera contain species capable of causing medically important accidents in humans: *Loxosceles, Phoneutria, Latrodectus* and *Atrax* [68]. In Brazil, *Loxosceles*, *Phoneutria* and *Latrodectus* are the most relevant genera and account for a large number of accidents in this country [69].

Spider venom contains a complex mixture of distinct compounds [70]. The main components are neurotoxins, proteins, peptides, enzymes, free amino acids and inorganic salts. Indeed, many toxins isolated from spider venom have been studied in relation to their role in ion channels [71] (Table 2).

**Table 2:** Examples of compounds from Brazilian spider venoms

Compounds	Examples	Species	Molecular Mass (kDa)	Action Mechanism	References
Neurotoxins	PnTx1,PnTx2, PnTx3	*Phoneutria nigriventer*	~6.0–9.0	Act on ion channels	72
	PnTx4	*Phoneutria nigriventer*	5.17	Inhibit reversible NMDA receptors in insects	73
	α-latrotoxin	*Latrodectus* sp*.*	~130	Influx of Ca^2+^ on presynaptic nerve endings	74
Enzymes	Phospholipase D	*Loxosceles* sp*.*	~31.0–32.0	Hydrolysis of membrane phospholipids	75
	(Sphingomyelinase)				
	Hyaluronidase	*Loxosceles* sp*.*	–	Catalyzes the hydrolysis of hyaluronan from the extracellular matrix	76
Proteinases	Metalloproteinase	*Loxosceles* sp*.*	~29.0	Lysis of the cell basement membrane	77
	Serinoproteinases	*Loxosceles* sp*.*	~85–95.0	Action on coagulation factors	78

These cocktails of substances that act by different pharmacological mechanisms have been extensively researched seeking to develop new drugs and biotechnological products [72].

The distinct characteristics of venom from each species determine its effect on humans in the event of an accident. Venom from the genus *Loxosceles*, or brown spider, has constituents such as hyaluronidases, metalloproteinases, phospholipases and other enzymes that provide a local effect with deep lesions, in contrast to the genus *Phoneutria*, whose venom produces neurotoxic activity [73]. The *Latrodectus* genus, or black widow spider, has neurotoxic venom components that act on presynaptic nerves of vertebrates [74].

In this review, we focused only on three genera responsible for the highest amount of medically important accidents in Brazil, *Loxosceles*, *Phoneutria* and *Latrodectus*, their principal components and respective contributions in physio-pharmacological studies.

### Biochemical characteristics of the venom from *Phoneutria*

Spiders of the *Phoneutria* genus are popularly known as “armed” due to the attack position they assume in a situation of danger. When these spiders face an opponent, they raise their front legs and lean on the back legs, presenting aggressive behavior [68].

The venom of this genus causes immediate and intense local pain radiating in the affected limb, but can progress into complications, especially in children and the elderly, such as salivation, sudoresis, hypertension, priapism and even death. These spiders are found in banana plants, palm trees and bromeliads. They are habitually nocturnal and responsible for most accident cases registered in Brazil. Such accidents occur mostly in the south and southeast regions of the country [75, 76].

Experimental studies have shown that the venom causes an activation of voltage-dependent sodium channels, and a blockade of voltage-dependent potassium and calcium channels in muscle fibers and sensory nerve endings in both the motor and autonomic nervous systems. As a consequence, there is a release of neurotransmitters, especially acetylcholine and catecholamines, which explains the following symptoms: severe pain at the bite site, sweating, agitation, salivation and, in severe cases, arrhythmias and priapism [75, 77, 78].

This venom is a cocktail consisting of peptides, free amino acids, histamine, serotonin and serine proteinases [79, 80]. Furthermore, the *Phoneutria nigriventer* venom is largely composed of neurotoxins.

The *Phoneutria* neurotoxins are similar to those from scorpion venoms. They present different amino acid sequences, but are rich in cysteines forming three or four disulfide bonds, which are responsible for peptide stability. In this genus, for example, there are three neurotoxins lethal to mice, denominated PnTx1, PnTx2 and PnTx3. The fraction PnTx4 modifies the neuromuscular response in insects [75, 79].

The PnTx2 fraction is composed of nine different peptides, which are mainly responsible for the overall effect of the venom. Of these nine peptides, the Tx2-5 and Tx2-6 are active in smooth muscle relaxation of the corpus cavernosum in rats and rabbits, causing erection [81–83]. This fact, along with the discovery that some of these fractions have insecticidal activity, has drawn the attention of researchers to the study and characterization of the *Phoneutria* venom.

In addition, PnTx4 was able to inhibit glutamate uptake by rat synaptosomes. The toxin Tx4(5–5), a polypeptide composed of 47 amino acid, displays a potent insecticidal activity. This toxin reversibly inhibited the N-methyl-D-aspartate (NMDA) subtype receptor [84].

A comparison of the proteomes of *P. nigriventer*, *P. reidyi* and *P. keyserlingi* revealed a large number of neurotoxic peptides that act on ion channels, which cause paralysis and death when injected in mice, as well as proteinases and peptides with insecticidal activity and non-toxic peptides [85].

Spiders contain innumerous peptides with interesting actions but with a low amount in the venom; for this reason, these components have been synthesized or cloned and expressed in bacteria or yeast. An example is a recombinant of PnTx-1 and PnTx3-4 from *Phoneutria nigriventer* venom. These studies open new perspectives in drug development and research [86, 87].

### Biochemical characteristics of the venom from *Loxosceles*

The different species of the genus *Loxosceles* are distributed globally. They are found in South America, North America, Europe, Africa, Oceania and Asia. They are popularly known as brown spiders and comprise more than 30 species in South America. In Brazil, the highest incidence of these spiders is in the southern and southeastern regions, where the *L. gaucho*, *L. laeta* and *L. intermedia* species are found [73, 88–90].

A brown spider bite can cause cutaneous or systemic (or both in some cases) manifestations in the victims. At least three actions of the loxoscelic venom are described: proteolysis with dermonecrosis at the bite site with a gravitational lesion; hemolytic action with intravascular hemolysis, which may lead to acute renal failure, and coagulant activity with thrombocytopenia, hypofibrinogenemia, prolongation of clotting time and disseminated intravascular coagulation [91, 92].

Brown spider venom is a mixture of toxins composed of proteins and also low-molecular-weight constituents. Numerous toxins have been identified and characterized biochemically. Among these are hydrolases, hyaluronidase, lipases, metallo—and serine proteinases, peptidases, collagenases, alkaline phosphatase and phospholipase or sphingomyelinase D [93–96].

The sphingomyelinases are phospholipases D considered the major components of the venom and are primarily responsible for dermonecrotic lesions. Furthermore, these enzymes are related to reactions involving components of the complement system, migration of polymorphonuclear leukocytes, platelets aggregation and inflammatory response [97].

Although sphingomyelinase D plays a key role in the *Loxosceles* envenoming and is the major component, studies have shown that the clinical manifestations are the result of an interaction between several other components in the venom [98].

Studies of *L. gaucho*, *L. deserta* and *L. reclusa* venom demonstrated the presence of metalloproteinases with gelatinolytic, caseinolytic and fibrinogenolytic activity. These enzymes appear to be involved with the signs and symptoms of envenoming. Some of these metalloproteinases present astacin-like activity. The astacins are zinc-dependent proteinases with such diverse functions as hydrolysis, digestion of peptides and degradation of extracellular matrix. These astacin-like metalloproteinases have been identified in the venom of *L. gaucho* and *L. laeta* [93, 95, 99, 100].

In addition, two serine proteinases from the same species of *Loxosceles* have been reported to hydrolyze gelatin [100, 101]. The authors concluded that the activity of serine proteinases complements other fibrinogenolytic proteinases in disseminated intravascular coagulation, triggered by loxoscelic venom [95, 101]. Furthermore, another enzyme that plays a key role in envenoming is hyaluronidase, which is responsible for the gravitational effect on the skin that spreads the venom [73, 95].

Toxins from *Loxosceles* venom have been cloned and expressed using cDNA. An example of recombinant protein generated by loxoscelic venom is *Loxosceles intermedia* recombinant dermonecrotic toxin (LiRecDT), which has properties similar to the *L. intermedia* venom, with respect to inflammatory and dermonecrotic activity, and stimulates nephrotoxicity in rats [73]. Furthermore, many sphingomyelinases have been cloned from the *Loxosceles* cDNA glands and expressed to obtain larger amounts of this enzyme and allow study of the structure and function of these toxins [97, 98].

### Biochemical characteristics of the venom from *Latrodectus* genus

Worldwide, more than 40 species of the genus *Latrodectus* are found in tropical and subtropical regions***.*** In Brazil, only three species occur: *L. geometricus*, *L. mactans* and *L. curacaviensis*, which inhabit mainly the northeast region [102, 103]. However, the presence of another specie, *L. mirabilis*, was recently described in the southern Brazilian state of Rio Grande do Sul [104].

The bites of these spiders, known as black widows, provoke clinical manifestations that include pain, hypertension, spasms, “facies latrodectismica”, vomiting, abdominal pain and muscle cramping. In severe cases, the patient may present myocardial infarction and compartment syndrome [102, 105].

The *Latrodecuts* venom contains a cocktail of substances, but its major component is α-latrotoxin (α-LTX), a neurotoxin that acts selectively on presynaptic nerve endings and provokes a discharge of neurotransmitters. This toxin is a protein with high molecular mass (about 130 kDa of mature toxin), but shows no enzymatic activity [74, 106–110].

The effects of the LTX seem to be related to the formation of pores in the membrane. LTX binds to specific receptors (named neurexin and latrophilin) which can facilitate the insertion of this toxin and subsequent influx of Ca^2+^ [106, 111, 112].

LTXs have targeted insects (latroinsectotoxins), crustaceans (latrocrustatoxin) and mammals. Many of these latrotoxins have been cloned and studied in relation to their structure, maturation and activity. Moreover, these toxins can help to elucidate the mechanisms of neurotransmitter release and to identify neuronal cell-surface receptors [113].

### Ticks

The known tickborne diseases are of great interest in the field of public health. Ticks are rarely considered venomous but some studies provide evidence to the contrary [5, 114–116]. Ticks, as vectors of disease transmission to humans, rank just behind mosquitoes as the most important arthropod transmitters of pathogens to several animal species [117]. Although these diseases have focal features on some regions, they have been recognized worldwide. Virus and bacteria are the main causes of the diseases transmitted by ticks. Among the virus-associated diseases, we can cite encephalitis, Crimean-Congo hemorrhagic fever, Omsk hemorrhagic fever, Colorado tick fever, Powassan encephalitis, Langat encephalitis and louping ill encephalitis. Some tickborne diseases associated with bacteria have already been described including tularemia, ehrlichiosis (monocytic and granulocity), rickettsiosis (spotted fever), Lyme borreliosis (Lyme disease) as well an infection caused by a protozoan, babesiosis [118–123].

Ticks are cosmopolitan and associated with numerous diseases besides being the most important group of ectoparasites of wild animals [118, 124]. Today, approximately 899 tick species have been described and distributed among three families: Ixodidae, Argasidae and Nuttalliellidae [118, 124–126]. There are several genera of ticks, most importantly *Ixodes*, *Dermacentor*, *Boophilus*, *Rhipicephalus*, *Haemaphysalis*, *Hyalomma* and *Amblyomma,* which belong to the family Ixodidae [126].

In Brazil, studies have reported the existence of 55 species, divided into six genera of the family Ixodidae (*Ixodes*, *Amblyomma*, *Haemaphysalis*, *Anocentor*, *Rhipicephalus* and *Boophilus*) and four genera of the Argasidae family (*Argas*, *Ornithodoros*, *Antricola* and *Otobius*). The Ixodidae family includes the most of the species of medical and veterinary importance in Brazil, where the genus *Amblyomma* (the largest genus containing 33 species) is the most important in the medical field. The species *Amblyomma cajennense, A. aureolatum* and *A. cooperi* stand out in relation to the transmission of spotted fever [127, 128].

**Fig. 4:**
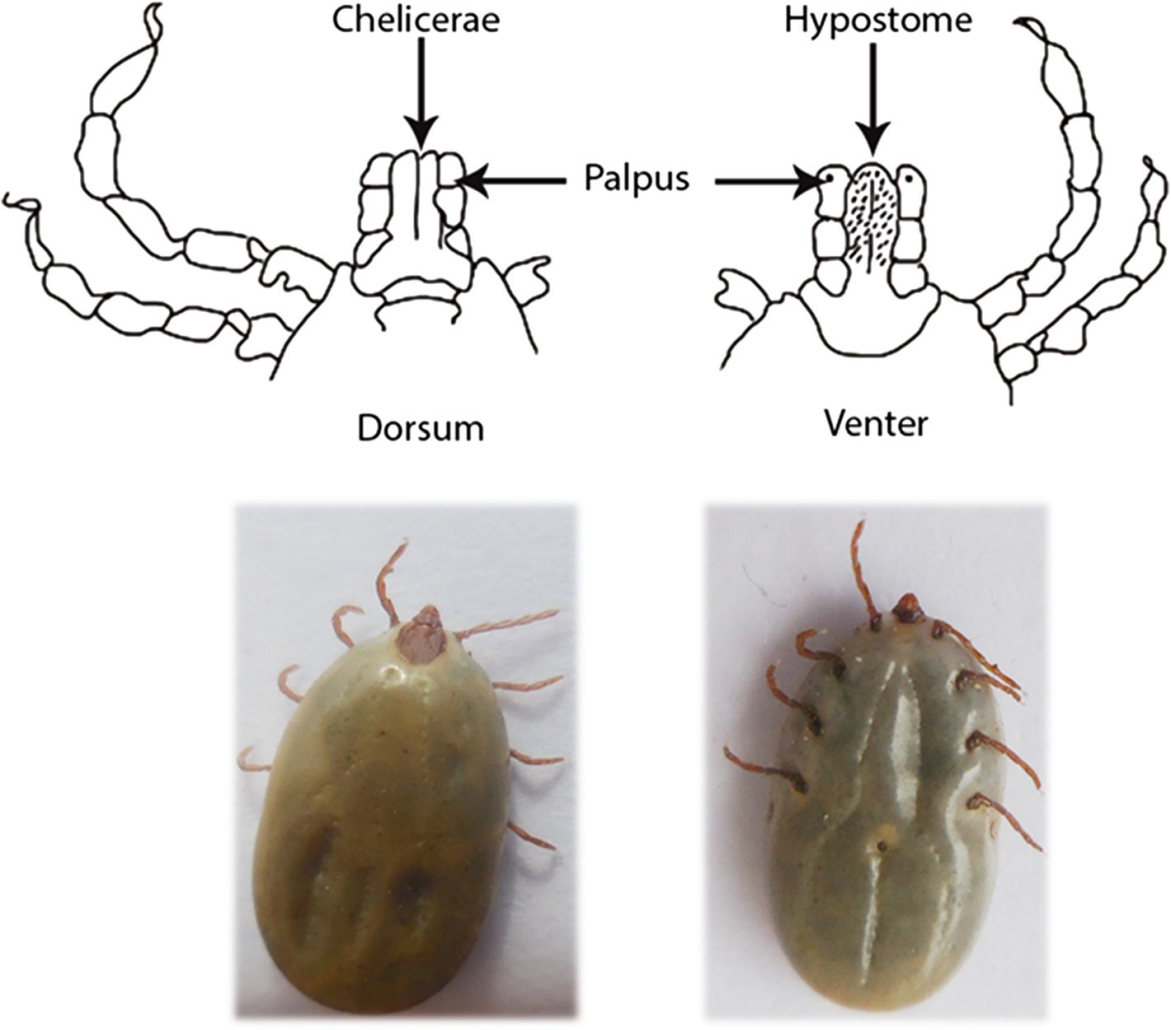
Photo of a tick and schematic representation of the *capitulum*. Dorsal and ventral morphology of the mouthpart of Ixodidae family ticks. On the dorsum it is possible to observe the chelicerae while the venter displays the hypostome. The palpus is observable on both sides (dorsum and venter). The hypostome is responsible for the dermal and epidermal damage (rupture of local blood vessels) during the tick’s feeding

Morphologically, ticks present two fused parts, namely the *capitulum* (or *gnathosoma*) that contains the head and mouthparts, and the *idiosoma* that contains the legs, digestive tract and reproductive organs (Fig. 4). The *capitulum* consists of three specialized structures: palpus, chelicerae and a hypostome. Nymph and adult ticks have eight legs whereas larval ticks possess six [118, 124, 129].

Several diseases can be transmitted during feeding by ticks, which are obligate hematophagous organisms. Dermal and epidermal damage (rupture of local blood vessels) are consequences of the insertion of the tick hypostome [125–127]. In contrast to the toxins of other arthropods such as scorpions and spiders, which utilize their toxins for protection as well as predation, the advantages of the tick toxins are still unclear and require additional research [130, 131]. We will discuss below the main compounds found in saliva from Brazilian families of ticks.

### Biochemical characteristics of tick saliva

Studies performed to evaluate the pharmacological complexity presented by hematophagous arthropods have shown that their saliva contains at least one anticlotting, one vasodilatory and one anti-platelet substance [132]. Among tick saliva components are descriptions of enzymes, enzyme inhibitors, host protein homologues, amine-binding lipocalins, immunoglobulin-binding proteins, receptor agonist/antagonist, calcium-binding components, cement cytokine components, cytokine expression modulators, non-proteinaceous bioactive components and other components related to cardiotoxic and neurotoxic factors [118, 119, 127, 130, 132, 133].

The *Amblyomma cajennense* is the most studied species in Brazil. After constructing a cDNA library on this tick, a serine protease Kunitz-type inhibitor was designed. This new inhibitor known as Amblyomin-X was able to decrease the number of metastatic events and the tumor mass in a B16F10 murine melanoma model by apoptosis induction [134–136]. Moreover, the Amblyomin-X was able to inhibit the factor Xa from coagulation cascade [136]. Although this species is the most studied in Brazil, most studies have focused on characterization and therapeutic application of Amblyomin-X [134–136].

Saliva-enzyme inhibitors have great biotechnological potential in the medical field. Ornithodorin (*Ornithodoros moubata*) and savignin (*Ornithodoros savignyi*) are examples of potent thrombin inhibitors from tick saliva [137, 138]. A novel tissue factor pathway inhibitor called ixolaris was found through the sialotranscriptome analysis of *I. scapularis* [139, 140]. Among the inhibitors of factor Xa, Salp14 is the main prototype identified in *I. scapularis* saliva, whereas tick anticoagulant peptide (TAP) is the main inhibitor of factor Xa from *Ornithodoros moubata* [141–144]. Variegin isolated from *Amblyomma variegatum* saliva is one of the smallest thrombin inhibitors (3.6 kDa) identified in nature. This inhibitor binds to thrombin with strong affinity and is considered an excellent model for the development of new inhibitors of this class [145].

In contrast to the scorpions, few neurotoxins were found in tick saliva to date. Some studies described neurotoxins such as HT-1 (holocyclotoxins) in the *Ixodes holocyclus* tick saliva and another still unnamed one in the *Rhipicephalus evertsi evertsi* tick saliva [127, 146, 147].

The gene coding of the HT-1 neurotoxin in the saliva of the tick *I. holocyclus* showed high homology with the gene coding scorpion neurotoxin [114, 146]. The study of this toxin may help elucidate the potentially fatal tick paralysis caused by this arthropod [127, 146–157].

The presence of the phospholipase A_2_ (PLA_2_) was observed in saliva from *Amblyomma americanum.* This enzyme is secreted in the tick-host interface, and probably plays an important role during prolonged tick feeding. The PLA_2_ does not contribute to the anticoagulant activities but is associated with hemolytic activity observed during feeding [158, 159].

Some lectins were characterized in the ticks *O. moubata* (Dorin M and OMFREP) and *I. ricinus* (ixoderin A and ixoderin B). Lectins play roles in the innate immunity of ticks whereas that of *R. microplus* induces immunosuppression in mice [5, 160–162].

An antimicrobial protein was identified in the hemolymph of the tick *Amblyomma hebraeum* and denominated hebraein (11 kDa). Native hebraein and its recombinant form, named hebraeinsin, revealed antimicrobial activities against the gram-positive and gram-negative bacteria (*S. aureus* and *E. coli*, respectively) and the fungus *Candida glabrata* [163]. In another study, two non-cationic defensin-like antimicrobial peptides, designated Amblyomma defensin peptide 1 and Amblyomma defensin peptide 2, were found in the *Amblyomma hebraeum* tick saliva [164]. The *Amblyomma* defensin peptide 2 showed antimicrobial activity against *E. coli* and *S. aureus*. Ixosin, another antimicrobial peptide, was isolated from salivary glands of the tick *Ixodes sinensis*. This peptide has 23 amino acids (without cysteine) and showed antimicrobial activity against *E. coli*, *S. aureus* and *C. albicans* [165]. Ixosin-B was purified and cloned from salivary glands of the *Ixodes sinensis* and showed antimicrobial activity against *E. coli*, *S. aureus* and *C. albicans* [166]. ISAMP, an antimicrobial peptide from *Ixodes scapularis* saliva, has a molecular weight of 5.3 kDa and exhibited antimicrobial activity against gram-negative and gram-positive bacteria. Additionally, it showed insignificant hemolytic action on rabbit red blood cells, suggesting that it is a safe antimicrobial peptide for possible use on mammals [167]. Table 3 summarizes the major components found in the tick saliva.

**Table 3:** Examples of compounds from tick saliva

Compounds	Examples	Species	Molecular Mass (kDa)^a^	Mechanism of Action	References
Enzyme Inhibitors	Amblyomin-X^b^	*Amblyomma cajennense*	15.0	Factor Xa Inhibition/induction of apoptosis in tumor cells	134–136
	Savignin	*Ornithodoros savignyi*	14.1	Thrombin inhibitor	137–138
	Ixolaris	*Ixodes scapularis*	18.4	Tissue factor pathway inhibitor	139–140
	Variegin	*Amblyomma variegatum*	3.6	Thrombin inhibitor	145
Neurotoxin	HT-1 (Holocyclotoxins)	*Ixodes holocyclus*	7.8	Unclear	114, 146–148
Enzyme	Phospholipase A_2_	*Amblyomma americanum*	55.7 ± 1.3	Hydrolysis of membrane phospholipids	158–159
Proteins	Hebraein	*Amblyomma hebraeum*	11.0	Unclear	163
	Ixosin	*Ixodes sinensis*	8.8	Unclear	165
	ISAMP	*Ixodes scapularis*	5.3	Unclear	167

^a^Data obtained from references and uniprot.org

^b^Compound found only in the transcriptome

After the identification of molecules with important pharmacological actions from natural sources, another possible alternative to obtain peptides is chemical synthesis. Zheng *et al.* [168] synthetized a defensin-like antimicrobial peptide obtained from a cDNA library of the male accessory glands of *Haemaphysalis longicornis*. This peptide, based on the predicted mature portion of HlMS-defensin, was tested against a variety of gram-positive and gram-negative bacteria and fungi, showing antimicrobial activity against all standard strains [168].

Defensins are small proteins present in vertebrates, invertebrates and plants and are responsible for their defense against several microorganisms. Two isoforms of the defensin gene, denominated def1 and def2, were found in saliva of *Ixodes ricinus* ticks; synthetic peptides from these defensins were tested against bacteria and yeast [169]. These defensins showed an antimicrobial activity against gram-positive bacteria, but were not effective against gram-negative ones or yeast [169]. Structurally, these defensins contain six cysteine residues and present as their main action mechanism cell membrane lysis by a formation of channels [169]. With the increasing number of microorganisms resistant to conventional antibiotics, the saliva of ticks is becoming an important source for the discovery of new compounds to treat several diseases.

## Conclusions

In this review we have highlighted the main biologically active components present in scorpion and spider venoms, as well as tick saliva, which are of great importance in the medical field in Brazil. We have also shown that the study of arachnid venoms and saliva provides numerous compounds with great biotechnological potential. The biochemical characterization of these compounds, combined with the advent of molecular biology techniques, enables the development of new biotechnological products with relevant applications. Additionally, this study allows the understanding of the physiological processes involved in the envenomings and diseases transmitted by ticks, thereby facilitating the obtainment of a more effective therapy.

## Abbreviations

α-NaScTx: α-neurotoxins with action on Na^+^ channels
α-KTx: α-neurotoxins with action on K^+^ channels
α-LTX: α-latrotoxin
β-NaScTx: β-neurotoxins with action on Na^+^ channels
β-KTxs: β-neurotoxins with action on K^+^ channels
γ-KTxs: γ-neurotoxins with action on K^+^ channels
κ-KTxs: κ-neurotoxins with action on K^+^ channels
AST: Aspartate aminotransferase
AVIT: Alanine-valine-isoleucine-threonine
CAP: Catabolite activator protein
CK: Creatine kinase
Kv: Voltage-gated K^+^ channels
LD: Lactate dehydrogenase
Nav: Voltage-gated Na^+^ channels
PLA_2_: Phospholipase A_2_

## References

[1] World Health Organization. Neglected tropical diseases: the 17 neglected tropical diseases. http://www.who.int/neglected_diseases/diseases/summary/en/

[2] Marcussi S, Arantes EC, Soares AM (2011). Escorpiões: biologia, envenenamento e mecanismos de ação de suas toxinas.

[3] Buchel W (1979). Acúleos que matam.

[4] Likes K, Banner W, Chavez M (1984). *Centruroides exilicauda* envenomation in Arizona. West J Med.

[5] Cabezas-Cruz A, Valdés JJ (2014). Are ticks venomous animals?. Front Zool.

[6] Regier JC, Shultz JW, Zwick A, Hussey A, Ball B, Werzer R (2010). Arthropod relationships revealed by phylogenomic analysis of nuclear protein-coding sequences. Nature.

[7] Bawaskar HS, Bawaskar PH (2012). Scorpion sting: update. J Assoc Phys India.

[8] Chippaux JP (2012). Emerging options for the management of scorpion stings. Drug Des Devel Ther.

[9] Portal Saúde: Acidentes por Escorpiões. http://portalsaude.saude.gov.br (2012).

[10] Warrell DA (2012). Venomous bites, stings, and poisoning. Infect Dis Clin North Am.

[11] Cologna CT, Marcussi S, Giglio JR, Soares AM, Arantes EC (2009). *Tityus serrulatus* scorpion venom and toxins: an overview. Protein Pept Lett.

[12] Hmed BN, Serria HT, Mounir ZK (2013). Scorpion peptides: potential use for new drug development. J Toxicol.

[13] Norwegian University of Science & Technology : The scorpion files. http://www.ntnu.no/ub/scorpion-files/.

[14] Bosmans F, Tytgat J (2007). Voltage-gated sodium channel modulation by scorpion alpha-toxins. Toxicon.

[15] Balozet L, Bucherl W, Buckley EE (1971). Scorpionism in the Old World. Venomous animals and their venoms.

[16] Reckziegel GC, Pinto VL (2014). Scorpionism in Brazil in the years 2000 to 2012. J Venom Anim Toxins incl Trop Dis.

[17] Dorce ALC, Dorce VA, Nencioni ALA (2014). Mild reproductive effects of the *Tityus bahiensis* scorpion venom in rats. J Venom Anim Toxins incl Trop Dis.

[18] Quintero-Hernández V, Jiménez-Vargas JM, Gurrola GB, Valdivia HH, Possani LD (2013). Scorpion venom components that affect ion-channels function. Toxicon.

[19] Possani LD, Becerril B, Delepierre M, Tytgat J (1999). Scorpion toxins specific for Na + −channels. Eur J Biochem.

[20] Watt DD, Simard JM (1984). Neurotoxic proteins in scorpion venom. J Toxicol Toxin Rev.

[21] Pucca MB, Amorim FG, Cerni FA, Bordon KDCF, Cardoso IA, Anjolette FAP (2014). Influence of post-starvation extraction time and prey-specific diet in *Tityus serrulatu*s scorpion venom composition and hyaluronidase activity. Toxicon.

[22] Oliveira FN, Mortari MR, Carneiro FP, Guerrero-Vargas JA, Santos DM, Pimenta A (2013). Another record of significant regional variation in toxicity of *Tityus serrulatus* venom in Brazil: a step towards understanding the possible role of sodium channel modulators. Toxicon.

[23] Rodríguez-Ravelo R, Coronas FI, Zamudio FZ, González-Morales L, López GE, Urquiola AR (2013). The Cuban scorpion *Rhopalurus junceus* (Scorpiones, Buthidae): component variations in venom samples collected in different geographical areas. J Venom Anim Toxins incl Trop Dis.

[24] Kalapothakis E, Chávez-Olórtegui C (1997). Venom variability among several *Tityus serrulatus* specimens. Toxicon.

[25] Tan PT, Veeramani A, Srinivasan KN, Ranganathan S, Brusic V (2006). SCORPION2: a database for structure-function analysis of scorpion toxins. Toxicon.

[26] Possani LD, Merino E, Corona M, Bolivar F, Becerril B (2000). Peptides and genes coding for scorpion toxins that affect ion-channels. Biochimie.

[27] Housset D, Habersetzer-Rochat C, Astier JP, Fontecilla-Camps JC (1994). Crystal structure of toxin II from the scorpion *Androctonus australis* Hector refined at 1.3 A resolution. J Mol Biol.

[28] Oren DA, Froy O, Amit E, Kleinberger-Doron N, Gurevitz M, Shaanan B (1998). An excitatory scorpion toxin with a distinctive feature: an additional α helix at the C terminus and its implications for interaction with insect sodium channels. Structure.

[29] Possani LD, Martin BM, Fletcher MD, Fletcher PL (1991). Discharge effect on pancreatic exocrine secretion produced by toxins purified from *Tityus serrulatus* scorpion venom. J Biol Chem.

[30] Kalapothakis E, Jardim S, Magalhães AC, Mendes TM, de Marco L, Afonso LC (2001). Screening of expression libraries using ELISA: identification of immunogenic proteins from *Tityus bahiensis* and *Tityus serrulatus* venom. Toxicon.

[31] Batista CV, Román-González SA, Salas-Castillo SP, Zamudio FZ, Gómez-Lagunas F, Possani LD (2007). Proteomic analysis of the venom from the scorpion *Tityus stigmurus*: biochemical and physiological comparison with other *Tityus* species. Comp Biochem Physiol C Toxicol Pharmacol.

[32] Teixeira CE, de Oliveira JF, Baracat JS, Priviero FB, Okuyama CE, Rodrigues Netto N (2004). Nitric oxide release from human corpus cavernosum induced by a purified scorpion toxin. Urology.

[33] Becerril B, Marangoni S, Possani LD (1997). Toxins and genes isolated from scorpions of the genus *Tityus*. Toxicon.

[34] Zoccal KF, Bitencourt Cda S, Secatto A, Sorgi CA, Bordon Kde C, Sampaio SV (2011). *Tityus serrulatus* venom and toxins Ts1, Ts2 and Ts6 induce macrophage activation and production of immune mediators. Toxicon.

[35] Tytgat J, Chandy KG, Garcia ML, Gutman GA, Martin-Eauclaire MF, van der Walt JJ (1999). A uniform nomenclature for short-chain peptides isolated from scorpion venoms: α - KT_x_ molecular subfamilies. Trends Pharmacol Sci.

[36] Corona M, Gurrola GB, Merino E, Cassulini RR, Valdez-Cruz NA, García FIV (2002). A large number of novel Ergtoxin-like genes and ERG K + −channels blocking peptides from scorpions of the genus *Centruroides*. FEBS Lett.

[37] Rodríguez de la Vega RC, Possani LD (2004). Current views on scorpion toxins specific for K + −channels. Toxicon.

[38] Mouhat S, Jouirou B, Mosbah A, de Waard M, Sabatier J (2004). Diversity of folds in animal toxins acting on ion channels. Biochem J.

[39] Chagot B, Pimentel C, Dai L, Pil J, Tytgat J, Nakajima T (2005). An unusual fold for potassium channel blockers: NMR structure of three toxins from the scorpion *Opisthacanthus madagascariensis*. Biochem J.

[40] Saucedo AL, Flores-Solis D, de la Vega RC R, Ramírez-Cordero B, Hernández-López R, Cano-Sánchez P (2012). New tricks of an old pattern structural versatility of scorpion toxins with common cysteine spacing. J Biol Chem.

[41] Giangiacomo KM, Garcia ML, McManus OB (1992). Mechanism of iberiotoxin block of the large-conductance calcium-activated potassium channel from bovine aortic smooth muscle. Biochemistry.

[42] Blaustein MP, Rogowski RS, Schneider MJ, Krueger BK (1991). Polypeptide toxins from the venoms of Old World and New World scorpions preferentially block different potassium channels. Mol Pharmacol.

[43] Papp F, Batista CV, Varga Z, Herceg M, Román-González SA, Gaspar R (2009). Tst26, a novel peptide blocker of Kv1.2 and Kv1.3 channels from the venom of *Tityus stigmurus*. Toxicon.

[44] Abdel-Mottaleb Y, Coronas FV, de Roodt AR, Possani LD, Tytgat JA (2006). A novel toxin from the venom of the scorpion *Tityus trivittatus*, is the first member of a new alpha-KTx subfamily. FEBS Lett.

[45] D’Suze G, Zamudio F, Gómez-Lagunas F, Possani LD (1999). A novel K^+^ channel blocking toxin from *Tityus discrepans* scorpion venom. FEBS Lett.

[46] Diego-García E, Abdel-Mottaleb Y, Schwartz EF, Rodríguez De L, Vega RC, Tytgat J (2008). Cytolytic and K^+^ channel blocking activities of beta-KTx and scorpine-like peptides purified from scorpion venoms. Cell Mol Life Sci.

[47] Diego-García E, Schwartz EF, D’Suze G, González SA, Batista CV, García BI (2007). Wide phylogenetic distribution of scorpine and long-chain beta-KTx-like peptides in scorpion venoms: identification of “orphan” components. Peptides.

[48] Wulff H, Castle NA, Pardo LA (2009). Voltage-gated potassium channels as therapeutic drug targets. Nat Rev Drug Discov.

[49] Verano-Braga T, Figueiredo-Rezende F, Melo MN, Lautner RQ, Gomes ER, Mata-Machado LT (2010). Structure-function studies of *Tityus serrulatus* Hypotensin-I (TsHpt-I): A new agonist of B(2) kinin receptor. Toxicon.

[50] Guo X, Ma C, Du Q, Wei R, Wang L, Zhou M (2013). Two peptides, TsAP-1 and TsAP-2, from the venom of the Brazilian yellow scorpion, *Tityus serrulatus*: evaluation of their antimicrobial and anticancer activities. Biochimie.

[51] Venancio EJ, Portaro FC, Kuniuoshi AK, Carvalho DC, Pidde-Queiroz G, Tambourgi DV (2013). Enzymatic properties of venoms from Brazilian scorpions of *Tityus* genus and the neutralisation potencial of therapeutical antivenoms. Toxicon.

[52] Almeida DD, Scortecci KC, Kobashi LS, Agnez-Lima LF, Medeiros SR, Silva-Junior AA (2012). Profiling the resting venom gland of the scorpion *Tityus stigmurus* through a transcriptomic survey. BMC Genomics.

[53] Alvarenga ER, Mendes TM, Magalhães BF, Siqueira FF, Dantas AE, Barroca TM (2012). Transcriptome analysis of the *Tityus serrulatus* scorpion venom gland. Open J Genetics.

[54] Flecther PL, Fletcher MD, Weninger K, Anderson TE, Martin BM (2009). Vesicle-associated membrane protein (VAMP) cleavage by a new metalloprotease from the Brazilian scorpion *Tityus serrulatus*. J Biol Chem.

[55] Ortiz E, Rendón-Anaya M, Rego SC, Schwartz EF, Possani LD (2013). Antarease-like Zn-metalloproteases are ubiquitous in the venom of different scorpion genera. Biochim Biophys Acta.

[56] Carmo AO, Oliveira-Mendes BB, Horta CC, Magalhães BF, Dantas AE, Chaves LM (2014). Molecular and functional characterization of metalloserrulases, new metalloproteases from the *Tityus serrulatus* venom gland. Toxicon.

[57] Almeida FM, Pimenta AM, de Figueiredo SG, Santoro MM, Martin-Eauclaire MF, Diniz CR (2002). Enzymes with gelatinolytic activity can be found in *Tityus bahiensis* and *Tityus serrulatus* venoms. Toxicon.

[58] Pukrittayakamee S, Warell DA, Desakorn V, McMichael AJ, White NJ, Bunnag D (1988). The hyaluronidase activities of some southeast Asian snake venoms. Toxicon.

[59] Pessini AC, Takao TT, Cavalheiro EC, Vichnewski W, Sampaio SV, Giglio JR (2001). A hyaluronidase from *Tityus serrulatus* scorpion venom: isolation, characterization and inhibition by flavonoids. Toxicon.

[60] D’Suze G, Schwartz EF, García-Gómez BI, Sevcik C, Possani LD (2009). Molecular cloning and nucleotide sequence analysis of genes from a cDNA library of the scorpion *Tityus discrepans*. Biochimie.

[61] Diego-García E, Batista CV, García-Gómez BI, Lucas S, Candido DM, Gómez-Lagunas F (2005). The Brazilian scorpion *Tityus costatus* Karsch: genes, peptides and function. Toxicon.

[62] Guerrero-Vargas JA, Mourão CB, Quintero-Hernández V, Possani LD, Schwartz EF (2012). Identification and phylogenetic analysis of *Tityus pachyurus* and *Tityus obscurus* novel putative Na + −channel scorpion toxins. PLoS One.

[63] Sabatier JM, Waard M, Kastin AJ (2013). Animal toxins in the world of modern biotechnology. Handbook of biologically active peptides.

[64] Mamelak AN, Rosenfeld S, Bucholz R, Raubitschek A, Nabors LB, Fiveash JB (2006). Phase I single-dose study of intracavitary-administered iodine-131-TM-601 in adults with recurrent high-grade glioma. J Clin Oncol.

[65] Soroceanu L, Gillespie Y, Khazaeli MB, Sontheimer H (1998). Use of chlorotoxin for targeting of primary brain tumors. Cancer Res.

[66] Watkins JB, Klaassen CD (2008). Properties and toxicities of animal venoms. Casarett & Doull’s toxicology: the basic science of poisons.

[67] Minton SA Jr. Venom diseases. 1 st ed. Springfield: Charles C. Thomas Publisher LTD; 1974.

[68] Lucas SM, Cardoso JLC, Haddad Junior V, França FS (2009). Aranhas de interesse médico no Brasil. Animais peçonhentos no Brasil: biologia, clínica e terapêutica dos acidentes.

[69] Cristiano MP, Cardoso DC, Raymundo MS (2009). Contextual analysis and epidemiology of spider bite in southern Santa Catarina state, Brazil. Trans R Soc Trop Med Hyg.

[70] Jackson H, Parks TN (1989). Spider toxins: recent applications in neurobiology. Ann Rev Neurosci.

[71] Rash LD, Hodgson WC (2002). Pharmacology and biochemistry of spider venoms. Toxicon.

[72] Nicholson GM, Graudins A, Wilson HI, Little M, Broady KW (2006). Arachnid toxinology in Australia: from clinical toxicology to potential applications. Toxicon.

[73] Senff-Ribeiro A, Henrique Da Silva P, Chaim OM, Gremski LH, Paludo KS, Bertoni Da Silveira R (2008). Biotechnological applications of brown spider (*Loxosceles* genus) venom toxins. Biotechnol Adv.

[74] Kiyatkin NI, Dulubova IE, Chekhovskaya IA, Grishin EV (1990). Cloning and structure of cDNA encoding α-latrotoxin from black widow spider venom. FEBS Lett.

[75] Antunes E, Málaque CMS, Cardoso JLC, Haddad Junior V, França FS (2009). Mecanismo de ação do veneno de *Phoneutria* e aspectos clínicos do foneutrismo. Animais peçonhentos no Brasil: biologia, clínica e terapêutica dos acidentes.

[76] Lucas SM (1988). Spiders in Brazil. Toxicon.

[77] de Lima ME, Borges MH, Verano-Braga T, Torres FS, Montandon GG, Cardoso FL (2010). Some arachnidan peptides with potential medical application. J Venom Anim Toxins incl Trop Dis.

[78] Teixeira CE, Corrado AP, de Nucci G, Antunes E (2004). Role of Ca^2+^ in vascular smooth muscle contractions induced by *Phoneutria nigriventer* spider venom. Toxicon.

[79] Rezende Júnior L, Cordeiro MN, Oliveira EB, Diniz CR (1991). Isolation of neurotoxic peptides from the venom of the “armed” spider *Phoneutria nigriventer*. Toxicon.

[80] Nunes KP, Costa-Gonçalves A, Lanza LF, Cortes SF, Cordeiro MN, Richardson M (2008). Tx2-6 toxin of the *Phoneutria nigriventer* spider potentiates rat erectile function. Toxicon.

[81] Andrade E, Villanova F, Borra P, Leite K, Troncone L, Cortez I (2008). Penile erection induced *in vivo* by a purified toxin from the Brazilian spider *Phoneutria nigriventer*. BJU Int.

[82] Leite KR, Andrade E, Ramos AT, Magnoli FC, Srougi M, Troncone LR (2012). *Phoneutria nigriventer* spider toxin Tx2-6 causes priapism and death: a histopathological investigation in mice. Toxicon.

[83] Jung AR, Choi YS, Piao S, Park YH, Shrestha KR, Jeon SH (2014). The effect of PnTx2-6 protein from *Phoneutria nigriventer* spider toxin on improvement of erectile dysfunction in a rat model of cavernous nerve injury. Urology.

[84] De Figueiredo SG, de Lima ME, Nascimento Cordeiro M, Diniz CR, Patten D, Halliwell RF (2001). Purification and amino acid sequence of a highly insecticidal toxin from the venom of the brazilian spider *Phoneutria nigriventer* which inhibits NMDA-evoked currents in rat hippocampal neurones. Toxicon.

[85] Richardson M, Pimenta AM, Bemquerer MP, Santoro MM, Beirão PSL, Lima ME (2006). Comparison of the partial proteomes of the venoms of Brazilian spiders of the genus *Phoneutria*. Comp Biochem Physiol C Toxicol Pharmacol.

[86] Silva AO, Peigneur S, Diniz MR, Tytgat J, Beirão PS (2012). Inhibitory effect of the recombinant *Phoneutria nigriventer* Tx1 toxin on voltage-gated sodium channels. Biochimie.

[87] Souza IA, Cino EA, Choy WY, Cordeiro MN, Richardson M, Chavez-Olortegui C (2012). Expression of a recombinant *Phoneutria* toxin active in calcium channels. Toxicon.

[88] Barbaro KC, Cardoso JLC, Cardoso JLC, França FS, Fan FW, Malaque CM, Haddad Junior V (2009). Mecanismo de ação do veneno de *Loxosceles* e aspectos clínicos do Loxoscelismo. Animais peçonhentos no Brasil: biologia, clínica e terapêutica dos acidentes.

[89] Machado LHA, Antunes MIPP, Mazini AM, Sakate M, Torres-Neto R, Fabris VE (2009). Necrotic skin lesion in a dog attributed to *Loxosceles* (brown spider) bite: a case report. J Venom Anim Toxins incl Trop Dis.

[90] Ramada JS, Becker-Finco A, Minozzo JC, Felicori LF, de Avila RA M, Molina F (2013). Synthetic peptides for *in vitro* evaluation of the neutralizing potency of *Loxosceles* antivenoms. Toxicon.

[91] Cardoso JLC, Schvartsman S (1992). Acidentes por *Loxosceles* (Loxoscelismo). Plantas venenosas e animais peçonhentos.

[92] Machado LF, Laugesen S, Botelho ED, Ricart CA, Fontes W, Barbaro KC (2005). Proteome analysis of brown spider venom: identification of loxnecrogin isoforms in *Loxosceles gaucho* venom. Proteomics.

[93] Feitosa L, Gremski W, Veiga SS, Elias MC, Graner E, Mangili OC (1998). Detection and characterization of metalloproteinases with gelatinolytic, fibronectinolytic and fibrinogenolytic activities in brown spider (*Loxosceles intermedia*) venom. Toxicon.

[94] Hogan CJ, Barbaro KC, Winkel K (2004). Loxoscelism: old obstacles, new directions. Ann Emerg Med.

[95] Barbaro KC, Sousa MV, Morhy L, Eickstedt VR, Mota I (1996). Compared chemical properties of dermonecrotic and lethal toxins from spiders of the genus *Loxosceles*(Araneae). J Protein Chem.

[96] da Silva PH, da Silveira RB, Appel MH, Mangili OC, Gremski W, Veiga SS (2004). Brown spiders and loxoscelism. Toxicon.

[97] Magalhães GS, Caporrino MC, Della-Casa MS, Kimura LF, Prezotto-Neto JP, Fukuda DA (2013). Cloning, expression and characterization of a phospholipase D from *Loxosceles gaucho* venom gland. Biochimie.

[98] da Silveira RB, Pigozzo RB, Chaim OM, Appel MH, Dreyfuss JL, Toma L (2006). Molecular cloning and functional characterization of two isoforms of dermonecrotic toxin from *Loxosceles intermedia* (brown spider) venom gland. Biochimie.

[99] Trevisan-Silva D, Gremski LH, Chaim OM, da Silveira RB, Meissner GO, Mangili OC (2010). Astacin-like metalloproteases are a gene family of toxins present in the venom of different species of the brown spider (genus *Loxosceles*). Biochimie.

[100] Trevisan-Silva D, Bednaski AV, Gremski LH, Chaim OM, Veiga SS, Senff-Ribeiro A (2013). Differential metalloprotease content and activity of three *Loxosceles* spider venoms revealed using two-dimensional electrophoresis approaches. Toxicon.

[101] Veiga SS, da Silveira RB, Dreyfus JL, Haoach J, Pereira AM, Mangili OC (2000). Identification of high molecular weight serine-proteases in *Loxosceles intermedia* (Brown spider) venom. Toxicon.

[102] Lira-da-Silva RM, Matos GB, Sampaio RO, Nunes TB (1995). Estudo retrospectivo de latrodectismo na Bahia Brasil. Rev Soc Bras Med Trop.

[103] Isbister GK, White J (2004). Clinical consequences of spider bites: recent advances in our understanding. Toxicon.

[104] Ott R, Rodrigues ENL, Marques MAL (2014). First record of *Lactrodectus mirabilis* (Araneae: Theridiidae) from southern Brazil and data on natural history of the species. Rev Colomb Entomol.

[105] Camp NE (2014). Black widow spider envenomation. J Emerg Nurs.

[106] Ushkaryov Y (2002). Alpha-latrotoxin: from structure to some functions. Toxicon.

[107] Grishin EV, Himmelreich NH, Pluzhnikov KA, Pozdnyakova NG, Storchak LG, Volkova TM (1993). Modulation of functional activities of the neurotoxin from black widow spider venom. FEBS Lett.

[108] Grishin EV (1998). Black widow spiders toxins: the present and the future. Toxicon.

[109] Rohou A, Nield J, Ushkaryov YA (2007). Insecticidal toxins from black widow spider venom. Toxicon.

[110] Danilevich VN, Grishin EV (2000). The cromossomal genes for black widow spider neurotoxins do not contain introns. Bioorg Khim.

[111] Ushkaryov YA, Petrenko AG, Geppert M, Sudhof TC (1992). Neurexins: synaptic cell surface proteins related to the α-latrotoxin receptor and laminin. Science.

[112] Lelianova VG, Davletov BA, Sterling A, Rahman MA, Grishin EV, Totty NF (1997). Alpha-latrotoxin receptor, latrophilin, is a novel member of the secretin family of G protein- coupled receptors. J Biol Chem.

[113] Ushkaryov YA, Volynski KE, Ashton AC (2004). The multiple actions of black widow spider toxins and their selective use in neurosecretion studies. Toxicon.

[114] Ishiwata K, Sasaki G, Ogawa J, Miyata T, Su ZH (2011). Phylogenetic relationships among insect orders based on three nuclear protein-coding gene sequences. Mol Phylogenet Evol.

[115] Low DH, Sunagar K, Undheim EA, Ali SA, Alagon AC, Ruder T (2013). Dracula’s children: molecular evolution of vampire bat venom. J Proteomics.

[116] Francischetti IM, Assumpção TC, Ma D, Li Y, Vicente EC, Uieda W (2013). The “Vampirome”: transcriptome and proteome analysis of the principal and accessory submaxillary glands of the vampire bat *Desmodus rotundus*, a vector of human rabies. J Proteomics.

[117] Parola P, Raoult D (2012). Ticks and tickborne bacterial diseases in humans: an emerging infectious threat. Clin Infect Dis.

[118] Anderson JF, Magnarelli LA (2008). Biology of ticks. Infect Dis Clin North Am.

[119] Organização Pan-Americana da Saúde (2004). Consulta de especialistas OPAS/OMS sobre Rickettioses nas Américas. Relatório Final.

[120] Estrada-Peña A, Hubálek Z, Rudolf I, Singh SK (2013). Tick-transmitted viruses and climate change. Viral Infections and Global Change.

[121] Lani R, Moghaddam E, Haghani A, Chang LY, AbuBakar S, Zandi K (2014). Tick-borne viruses: a review from the perspective of therapeutic approaches. Ticks Tick Borne Dis.

[122] Lantos PM, Wormser GP (2014). Chronic coinfections in patients diagnosed with chronic lyme disease: a systematic literature review. Am J Med.

[123] Greca H, Langoni H, Souza LC (2008). Brazilian spotted fever: a reemergent zoonosis. J Venom Anim Toxins incl Trop Dis.

[124] Estrada-Peña A, de la Fuente J (2014). The ecology of ticks and epidemiology of tick-borne viral diseases. Antiviral Res.

[125] Barker SC, Murrel A (2002). Phylogeny, evolution and historical zoogeography of ticks: a review of recent progress. Exp Appl Acarol.

[126] Mans BJ, Neitz AW (2004). Adaptation of ticks to a blood-feeding environment: evolution from a functional perspective. Insect Biochem Mol Biol.

[127] Steen NA, Barker SC, Alewood PF (2006). Proteins in the saliva of the Ixodida (ticks): Pharmacological features and biological significance. Toxicon.

[128] Cohen SB, Freye JD, Dunlap BG, Dunn JR, Jones TF, Moncayo AC (2010). Host associations of *Dermacentor*, *Amblyomma*, and *Ixodes* (Acari: Ixodidae) ticks in Tennessee. J Med Entomol.

[129] Buczek A, Olszewski K, Andrearczyk A, Zwoliński J (2004). Morphology of tick tarsus (Acari: Ixodia) modifications connected with life cycle, behavior and habitat. Wiad Parazytol.

[130] Mans BJ, Steinmann CM, Venter JD, Louw AI, Neitz AW (2002). Pathogenic mechanisms of sand tampan toxicoses induced by the tick Ornithodoros savignyi. Toxicon.

[131] Mans BJ, Gothe R, Neitz AW (2014). Biochemical perspectives on paralysis and other forms of toxicoses caused by ticks. Parasitology.

[132] Ribeiro JM, Francischetti IM (2003). Role of arthropod saliva in blood feeding: sialome and post-sialome perspectives. Annu Rev Entomol.

[133] Campbell F, Atwell R, Fenning A, Hoey A, Brown L (2004). Cardiovascular effects of the toxin(s) of the Australian paralysis tick, *Ixodes holocyclus*, in the rat. Toxicon.

[134] Simons SM, De-Sá-Júnior PL, Faria F, Batista IF, Barros-Battesti DM, Labruna MB (2011). The action of *Amblyomma cajennense* tick saliva in compounds of the hemostatic system and cytotoxicity in tumor cell lines. Biomed Pharmacother.

[135] Drewes CC, Dias RY, Hebeda CB, Simons SM, Barreto SA, Ferreira Junior JM (2012). Actions of the Kunitz-type serine protease inhibitor Amblyomin-X on VEGF-A-induced angiogenesis. Toxicon.

[136] Chudzinski-Tavassi AM, De-Sá-Júnior PL, Simons SM, Maria DA, de Souza Ventura J, Batista IF (2010). A new tick Kunitz type inhibitor, Amblyomin-X, induces tumor cell death by modulating genes related to the cell cycle and targeting the ubiquitin-proteasome system. Toxicon.

[137] Nienaber J, Gaspar AR, Neitz AW (1999). Savignin, a potent thrombin inhibitor isolated from the salivary glands of the tick *Ornithodoros savignyi* (Acari: Argasidae). Exp Parasitol.

[138] Mans BJ, Louw AI, Neitz AW (2002). Evolution of hematophagy in ticks: common origins for blood coagulation and platelet aggregation inhibitors from soft ticks of the genus *Ornithodoros*. Mol Biol Evol.

[139] Francischetti IM, Valenzuela JG, Andersen JF, Mather TN, Ribeiro JM (2002). Ixolaris, a novel recombinant tissue factor pathway inhibitor (TFPI) from the salivary gland of the tick, *Ixodes scapularis:* identification of factor X and factor Xa as scaffolds for the inhibition of factor VIIa/tissue factor complex. Blood.

[140] Ribeiro JM, Alarcon-Chaidez F, Francischetti IM, Mans BJ, Mather TN, Valenzuela JG (2006). An annotated catalog of salivary gland transcripts from *Ixodes scapularis* ticks. Insect Biochem Mol Biol.

[141] Narasimhan S, Koski RA, Beaulieu B, Anderson JF, Ramamoorthi N, Kantor F (2002). A novel family of anticoagulants from the saliva of *Ixodes scapularis*. Insect Mol Biol.

[142] Waxman L, Smith DE, Arcuri KE, Vlasuk GP (1990). Tick anticoagulant peptide (TAP) is a novel inhibitor of blood coagulation factor Xa. Science.

[143] Van de Locht A, Stubbs MT, Bode W, Friedrich T, Bollschweiler C, Höffken W (1996). The ornithodorin-thrombin crystal structure, a key to the TAP enigma?. EMBO J.

[144] Lim-Wilby MS, Hallenga K, de Mayer M, Lasters I, Vlasuk GP, Brunck TK (1995). NMR structure determination of tick anticoagulant peptide (TAP). Protein Sci.

[145] Koh CY, Kazimirova M, Trimnell A, Takac P, Labuda M, Nuttall PA (2007). Variegin, a novel fast and tight binding thrombin inhibitor from the tropical bont tick. J Biol Chem.

[146] Masina S, Broady KW (1999). Tick paralysis: development of a vaccine. Int J Parasitol.

[147] Crause JC, Verschoor JA, Coetzee J, Hoppe HC, Taljaard JN, Gothe R (1993). The localization of a paralysis toxin in granules and nuclei of prefed female *Rhipicephalus evertsi evertsi* tick salivary gland cells. Exp Appl Acarol.

[148] Hall-Mendelin S, Craig SB, Hall RA, O’Donoghue P, Atwell RB, Tulsiani SM (2011). Tick paralysis in Australia caused by *Ixodes holocyclus* Neumann. Ann Trop Med Parasitol.

[149] Almeida RAMB, Ferreira MA, Barraviera B, Haddad V (2012). The first reported case of human tick paralysis in Brazil: a new induction pattern by immature stages. J Venom Anim Toxins incl Trop Dis.

[150] Vink S, Daly NL, Steen N, Craik DJ, Alewood PF (2014). Holocyclotoxin-1, a cystine knot toxin from *Ixodes holocyclus*. Toxicon.

[151] Brazier I, Kelman M, Ward MP (2014). The association between landscape and climate and reported tick paralysis cases in dogs and cats in Australia. Vet Parasitol.

[152] Taraschenko OD, Powers KM (2014). Neurotoxin-induced paralysis: a case of tick paralysis in a 2-year-old child. Pediatr Neurol.

[153] Pecina CA (2012). Tick paralysis. Semin Neurol.

[154] Purwar S (2009). Tick paralysis: an uncommon dimension of tick-borne diseases. South Med J.

[155] Edlow JA, McGillicuddy DC (2008). Tick paralysis. Infect Dis Clin North Am.

[156] Schull DN, Litster AL, Atwell RB (2007). Tick toxicity in cats caused by *Ixodes* species in Australia: a review of published literature. J Feline Med Surg.

[157] Vedanarayanan V, Sorey WH, Subramony SH (2004). Tick paralysis. Semin Neurol.

[158] Bowman AS, Gengler CL, Surdick MR, Zhu K, Essenberg RC, Sauer JR (1997). A novel phospholipase A2 activity in saliva of the lone star tick, *Amblyomma americanum* (L). Exp Parasitol.

[159] Zhu K, Bowman AS, Dillwith JW, Sauer JR (1998). Phospholipase A2 activity in salivary glands and saliva of the lone star tick (Acari: Ixodidae) during tick feeding. J Med Entomol.

[160] Rego RO, Kovár V, Kopácek P, Weise C, Man P, Sauman I (2006). The tick plasma lectin, Dorin M, is a fibrinogen-related molecule. Insect Biochem Mol Biol.

[161] Bautista-Garfias CR, Martínez-Cruz MA, Córdoba-Alva F (1997). Lectin activity from the cattle tick (*Boophilus microplus*) saliva. Rev Latinoam Microbiol.

[162] Rego RO, Hajdusek O, Kovár V, Kopácek P, Grubhoffer L, Hypsa V (2005). Molecular cloning and comparative analysis of fibrinogen-related proteins from the soft tick *Ornithodoros moubata* and the hard tick *Ixodes ricinus*. Insect Biochem Mol Biol.

[163] Lai R, Takeuchi H, Lomas LO, Jonczy J, Rigden DJ, Rees HH (2004). A new type of antimicrobial protein with multiple histidines from the hard tick Amblyomma hebraeum. FASEB J.

[164] Lai R, Lomas LO, Jonczy J, Turner PC, Rees HH (2004). Two novel non-cationic defensin-like antimicrobial peptides from haemolymph of the female tick Amblyomma hebraeum. Biochem J.

[165] Yu D, Sheng Z, Xu X, Li J, Yang H, Liu Z (2006). A novel antimicrobial peptide from salivary glands of the hard tick Ixodes sinensis. Peptides.

[166] Liu Z, Liu H, Liu X, Wu X (2008). Purification and cloning of a novel antimicrobial peptide from salivary glands of the hard tick, *Ixodes sinensis*. Comp Biochem Physiol B Biochem Mol Biol.

[167] Pichu S, Ribeiro JM, Mather TN (2009). Purification and characterization of a novel salivary antimicrobial peptide from the tick Ixodes scapularis. Biochem Biophys Res Commun.

[168] Zheng H, Zhou L, Yang X, Wang D, Liu J (2012). Cloning and characterization of a male-specific defensin-like antimicrobial peptide from the tick *Haemaphysalis longicornis*. Dev Comp Immunol.

[169] Chrudimská T, Slaninová J, Rudenko N, Růžek D, Grubhoffer L (2011). Functional characterization of two defensing isoforms of the hard tick *Ixodes ricinus*. Parasit Vectors.

